# Supplementary material for: Longitudinal trajectories of blood glucose and 30-day mortality in patients with diabetes mellitus combined with acute myocardial infarction: A retrospective cohort analysis of the MIMIC database
Source: PLoS One. 2024 Sep 13;19(9):e0307905. doi: 10.1371/journal.pone.0307905 (PMC11398677; doi:10.1371/journal.pone.0307905)
Supplement: S1 Table — (DOCX) [file pone.0307905.s001.docx]

Table S1 Determination of the number of classes in LGMM model

| Number of classes | Log likelihood | AIC | BIC | Entropy | Class 1 proportion | Class 2 proportion | Class 3 proportion | Class 4 proportion | Class 5 proportion |
| --- | --- | --- | --- | --- | --- | --- | --- | --- | --- |
| 1 | -59658.11 | 119330 | 119367.5 | 1 | 100% |  |  |  |  |
| 2 | -58778.12 | 117580.2 | 117644.2 | 0.759 | 77.02% | 22.98% |  |  |  |
| 3 | -58345.38 | 116724.8 | 116815.3 | 0.845 | 75.90% | 10.97% | 13.13% |  |  |
| **4** | **-58133.19** | **116320.4** | **116464.3** | **0.818** | **72.09%** | **15.43%** | **9.19%** | **3.28%** |  |
| 5 | -58204.91 | 116453.8 | 116571.1 | 0.827 | 71.31% | 10.57% | 13.85% | 1.05% | 3.22% |

LGMM: latent growth mixture modeling, AIC: Akaike information criterion, BIC: Bayesian information criterion.
