# Supplementary material for: Longitudinal trajectories of blood glucose and 30-day mortality in patients with diabetes mellitus combined with acute myocardial infarction: A retrospective cohort analysis of the MIMIC database
Source: PLoS One. 2024 Sep 13;19(9):e0307905. doi: 10.1371/journal.pone.0307905 (PMC11398677; doi:10.1371/journal.pone.0307905)
Supplement: S2 Table — (DOCX) [file pone.0307905.s002.docx]

Table S2 Average posterior probability of the selected classes in LGMM model

| Classes | Probability 1 | Probability 2 | Probability 3 | Probability 4 |
| --- | --- | --- | --- | --- |
| 1 | **0.9306** | 0.0582 | 0.0102 | 0.0010 |
| 2 | 0.0838 | **0.8296** | 0.0556 | 0.0310 |
| 3 | 0.0427 | 0.0615 | **0.8940** | 0.0018 |
| 4 | 0.0044 | 0.0924 | 0.0172 | **0.8860** |

LGMM: latent growth mixture modeling.
