# Supplementary material for: Longitudinal trajectories of blood glucose and 30-day mortality in patients with diabetes mellitus combined with acute myocardial infarction: A retrospective cohort analysis of the MIMIC database
Source: PLoS One. 2024 Sep 13;19(9):e0307905. doi: 10.1371/journal.pone.0307905 (PMC11398677; doi:10.1371/journal.pone.0307905)
Supplement: S3 Table — (DOCX) [file pone.0307905.s003.docx]

Table S3 Covariates associated with 30-day mortality in AMI patients with DM

| Variables | HR (95% CI) | *P* |
| --- | --- | --- |
| Age | 1.28 (1.07-1.53) | **0.006** |
| Gender |  |  |
| Female | Ref |  |
| Male | 0.83 (0.59-1.17) | 0.292 |
| Race |  |  |
| Others | Ref |  |
| White | 0.96 (0.68-1.34) | 0.812 |
| Insurance |  |  |
| Medicare | Ref |  |
| Other | 0.87 (0.62-1.23) | 0.437 |
| Marital status |  |  |
| Married | Ref |  |
| Other/unknown | 1.12 (0.80-1.56) | 0.511 |
| AMI types |  |  |
| NSTEMI | Ref |  |
| STEMI | 3.24 (2.20-4.77) | **<0.001** |
| Unknown | 2.08 (1.05-4.12) | **0.035** |
| Urine output | 0.43 (0.34-0.55) | **<0.001** |
| Sepsis |  |  |
| No | Ref |  |
| Yes | 3.82 (2.73-5.34) | **<0.001** |
| CS |  |  |
| No | Ref |  |
| Yes | 5.65 (4.02-7.94) | **<0.001** |
| Weight | 1.07 (0.92-1.25) | 0.378 |
| HR | 1.30 (1.12-1.51) | **<0.001** |
| SBP | 0.94 (0.79-1.12) | 0.483 |
| DBP | 1.22 (1.06-1.41) | **0.006** |
| RR | 1.47 (1.34-1.62) | **<0.001** |
| Temperature | 1.01 (0.84-1.20) | 0.955 |
| SpO_2_ | 0.70 (0.63-0.77) | **<0.001** |
| SOFA | 1.36 (1.18-1.57) | **<0.001** |
| SAPS-II | 2.17 (1.89-2.48) | **<0.001** |
| GCS | 1.19 (0.96-1.47) | 0.120 |
| CCI | 1.90 (1.63-2.21) | **<0.001** |
| WBC | 1.34 (1.23-1.47) | **<0.001** |
| Platelet | 1.37 (1.20-1.57) | **<0.001** |
| HB | 1.15 (0.98-1.35) | 0.086 |
| RDW | 1.42 (1.26-1.59) | **<0.001** |
| Cr | 1.30 (1.19-1.42) | **<0.001** |
| INR | 1.26 (1.15-1.38) | **<0.001** |
| PT | 1.30 (1.18-1.43) | **<0.001** |
| BUN | 1.49 (1.36-1.65) | **<0.001** |
| Bicarbonate | 0.61 (0.54-0.69) | **<0.001** |
| Na | 1.14 (0.98-1.33) | 0.083 |
| K | 1.14 (0.97-1.33) | 0.102 |
| Chloride | 0.70 (0.61-0.81) | **<0.001** |
| Mg | 0.63 (0.52-0.77) | **<0.001** |
| Ventilation use |  |  |
| No | Ref |  |
| Yes | 1.11 (0.52-2.38) | 0.782 |
| Vasopressor use |  |  |
| No | Ref |  |
| Yes | 1.93 (1.24-2.99) | **0.003** |
| PCI/CABG |  |  |
| No | Ref |  |
| Yes | 0.15 (0.10-0.21) | **<0.001** |
| Thrombolysis use |  |  |
| No | Ref |  |
| Yes | 0.00 (0.00-Inf) | 0.993 |
| Insulin use |  |  |
| No | Ref |  |
| Yes | 0.35 (0.18-0.66) | **0.001** |
| Antiplatelet agents use |  |  |
| No | Ref |  |
| Yes | 2.01 (0.50-8.10) | 0.328 |
| Anticoagulation agents use |  |  |
| No | Ref |  |
| Yes | 4.02 (2.73-5.92) | **<0.001** |
| Statins use |  |  |
| No | Ref |  |
| Yes | 0.25 (0.17-0.37) | **<0.001** |

AMI: acute myocardial infarction, DM: diabetes mellitus, HR: hazard ratio, CI: confidence interval, Ref: reference, NSTEMI: Non-ST elevation myocardial infarction, STEMI: ST elevation myocardial infarction, CS: cardiogenic shock, HR: heart rate, SBP: systolic blood pressure, DBP: diastolic blood pressure, RR: respiratory rate, SOFA: the Sequential Organ Failure Assessment, SAPS: the Simplified Acute Physiology Score, GCS: the Glasgow Coma Scale, CCI: the Charlson Comorbidity Index, WBC: white blood cell, HB: hemoglobin, RDW: red cell distribution width, Cr: creatinine, INR: international normalized ratio, PT: prothrombin time, BUN: blood urea nitrogen, Na: sodium, K: potassium, Mg: magnesium, PCI: percutaneous coronary intervention, CABG: coronary artery bypass graft.
