# Supplementary material for: Longitudinal trajectories of blood glucose and 30-day mortality in patients with diabetes mellitus combined with acute myocardial infarction: A retrospective cohort analysis of the MIMIC database
Source: PLoS One. 2024 Sep 13;19(9):e0307905. doi: 10.1371/journal.pone.0307905 (PMC11398677; doi:10.1371/journal.pone.0307905)
Supplement: Attachment — Submitted filename: PONE review - DC.docx [file pone.0307905.s005.docx]

Basal characteristics of the patients in each longitudinal trajectory of blood glucose class were very different. It is possible that there were relevant non-measured variables that could introduce confounding. Although the potential for confounding was addressed, it should be highlighted and discussed further.

The blood glucose reduction in class 3 was associated with better outcomes, and the authors conjecture that this was owed to a better glucose-lowering treatment. But there is no data about the received treatment, so this trajectory could have other causes. Therefore, the conclusion should be more cautious in suggesting faster and stricter glucose control. While several studies have shown the benefits of managing hyperglycemia (>200 mg/dL), the risk of hypoglycemia-related events when using intensive insulin therapy should not be neglected.

It would be useful for a non-statistician to have a clear explanation in the manuscript about latent growth mixture modeling and how it derives trajectory classes from the data.
